# Scar heterogeneity on cardiovascular magnetic resonance as a predictor of appropriate implantable cardioverter defibrillator therapy

**DOI:** 10.1186/1532-429X-15-31

**Published:** 2013-04-10

**Authors:** Hussein Rayatzadeh, Alex Tan, Raymond H Chan, Shalin J Patel, Thomas H Hauser, Long Ngo, Jaime L Shaw, Susie N Hong, Peter Zimetbaum, Alfred E Buxton, Mark E Josephson, Warren J Manning, Reza Nezafat

**Affiliations:** 1 Department of Medicine, Boston, MA, USA; 2 Radiology, Beth Israel Deaconess Medical Center and Harvard Medical School, Boston, MA, USA; 3 Beth Israel Deaconess Medical Center, 330 Brookline Ave, Boston, MA 02215, USA

**Keywords:** Cardiovascular magnetic resonance, Late gadolinium enhancement, Implantable cardioverter defibrillator, Scar heterogeneity

## Abstract

**Background:**

Despite the survival benefit of implantable-cardioverter-defibrillators (ICDs), the vast majority of patients receiving an ICD for primary prevention do not receive ICD therapy. We sought to assess the role of heterogeneous scar area (HSA) identified by late gadolinium enhancement cardiovascular magnetic resonance (LGE-CMR) in predicting appropriate ICD therapy for primary prevention of sudden cardiac death (SCD).

**Methods:**

From September 2003 to March 2011, all patients who underwent primary prevention ICD implantation and had a pre-implantation LGE-CMR were identified. Scar size was determined using thresholds of 4 and 6 standard deviations (SD) above remote normal myocardium; HSA was defined using 3 different criteria; as the region between 2 SD and 4 SD (HSA_2-4SD_), between 2SD and 6SD (HSA2-6SD), and between 4SD and 6SD (HSA_4-6SD_). The end-point was appropriate ICD therapy.

**Results:**

Out of 40 total patients followed for 25 ± 24 months, 7 had appropriate ICD therapy. Scar size measured by different thresholds was similar in ICD therapy and non-ICD therapy groups (P = NS for all). However, HSA_2-4SD_ and HSA_4-6SD_ were significantly larger in the ICD therapy group (P = 0.001 and P = 0.03, respectively). In multivariable model HSA_2-4SD_ was the only significant independent predictor of ICD therapy (HR = 1.08, 95%CI: 1.00-1.16, P = 0.04). Kaplan-Meier analysis showed that patients with greater HSA_2-4SD_ had a lower survival free of appropriate ICD therapy (P = 0.026).

**Conclusions:**

In primary prevention ICD implantation, LGE-CMR HSA identifies patients with appropriate ICD therapy. If confirmed in larger series, HSA can be used for risk stratification in primary prevention of SCD.

## Background

Sudden cardiac death (SCD) accounts for 5.6-15% of annual mortality in the United States and industrialized countries [1] and is a major cause of mortality in patients with advanced heart failure and coronary heart disease. Implantable cardioverter-defibrillators (ICD) have been found to significantly reduce arrhythmic death in this population [2]. Based on the MADIT-II and SCD-HeFT trials [3], current guidelines recommend ICD implantation as a class I indication for primary prevention of SCD in patients with a left ventricular ejection fraction (LVEF) ≤ 30% as well as those with LVEF ≤ 35% that are New York Heart Association (NYHA) heart failure class II or III [4].

Current guidelines use LVEF as the major risk stratifier for primary prevention ICD implantation [4]. While efficacious, the majority of patients receiving an ICD for primary prevention do not utilize this expensive therapy [5]. On the other hand, many patients with an LVEF > 35% may develop potentially lethal ventricular arrhythmias (e.g. ventricular tachycardia and ventricular fibrillation) and SCD. Thus, there has been a growing focus on risk stratifying the patients at risk of SCD and finding the major predictors of SCD that play a role either independently or in conjunction with LVEF [6-8].

Multiple mechanisms underlie ventricular arrhythmias. Much evidence suggests a link between ventricular scar and arrhythmogenicity [9-12]. Late gadolinium enhancement cardiovascular magnetic resonance (LGE-CMR) can accurately characterize areas of myocardial injury and scar [13,14]. In one study of patients with LV dysfunction, infarct tissue heterogeneity on LGE-CMR was the only significant predictor of inducible sustained monomorphic VT [15]. However, VT inducibility is a surrogate end-point and may not reflect clinical efficacy. In the present study, we sought to investigate the correlation between LGE-CMR tissue heterogeneity and the occurrence of appropriate ICD therapy.

## Methods

### Study design and patient selection

The Beth Israel Deaconess Medical Center clinical CMR database was queried to identify all patients undergoing ICD implantation for primary SCD prevention from September 2003 to March 2011 who also had a pre-implantation CMR. Patients with ischemic and idiopathic non-ischemic cardiomyopathies were included. All patients with hypertrophic, inflammatory, infiltrative, and arrhythmogenic right ventricular cardiomyopathies were excluded. Patient demographics and clinical follow-up records from the hospital electronic medical records were reviewed.

The study was carried out with Beth Israel Deaconess Medical Center Institutional Review Board approval and written informed consent was waived. The authors had full access to the data and take responsibility for its integrity. All authors have read and agreed to the manuscript as written.

### Cardiovascular magnetic resonance

CMR was performed on a Philips 1.5 T (Philips Medical Systems, Amsterdam, Netherlands) CMR scanner with a commercial 5-element cardiac-surface coil. Cine images were acquired in a contiguous LV short-axis orientation with an electrocardiography-gated, breath-hold, steady-state free-precession sequence with full LV coverage (8-mm slice thickness, 2-mm interslice gap, in-plane spatial resolution 2 × 2 mm, 30 ms temporal resolution). LGE-CMR was performed 15 minutes after the intravenous administration of 0.2 mmol/kg gadolinium-DTPA (Magnevist; Schering, Berlin, Germany) with a 2-dimensional breath-hold, segmented inversion-recovery sequence (inversion time optimized by the Look-Locker sequence [inversion time scout] to null normal myocardium) acquired in the same orientation (short-axis stack) as the cine images, with the following imaging parameters: 8-mm slice thickness, 2-mm interslice gap, repetition time (TR) 4.2 ms, echo time (TE) 1.8 ms, flip angle 20°, field of view 320 × 320 mm^2^, matrix 160 × 160, and spatial resolution 2 × 2 mm^2^. In 9 patients LGE was performed using a 3D phase sensitive inversion recovery sequence (PSIR) with the following imaging parameters 10-mm slice thickness, 5-mm spacing between slices, TR 4.2 ms, TE 1.8 ms, flip angle 15°, field of view 320 × 320 mm^2^, matrix size 176 × 156, and spatial resolution 1.8 × 2.0 mm^2^.

### CMR analysis

CMR analyses were performed with commercially available software (QMassMR version 7.1.0; MedisInc, Leiden, Netherlands). LV endocardial and epicardial borders on both cine and LGE images were measured by planimetry, with special care taken to exclude papillary muscles and the intertrabecular blood pool. Left and right ventricular volume, mass, and ejection fraction were measured from the cine short-axis images using standard techniques [16].

The LV short-axis stack of LGE images was first assessed visually for the presence of LGE by two independent, experienced readers and disagreements were adjudicated by a senior observer. The readers were blinded to the outcome data.

The mean grayscale signal intensity (SI) and standard deviation (SD) for normal LV myocardium were measured for each patient, with a region of interest placed in a portion of nulled myocardium (i.e., without LGE on visual inspection).

**Figure 1:**
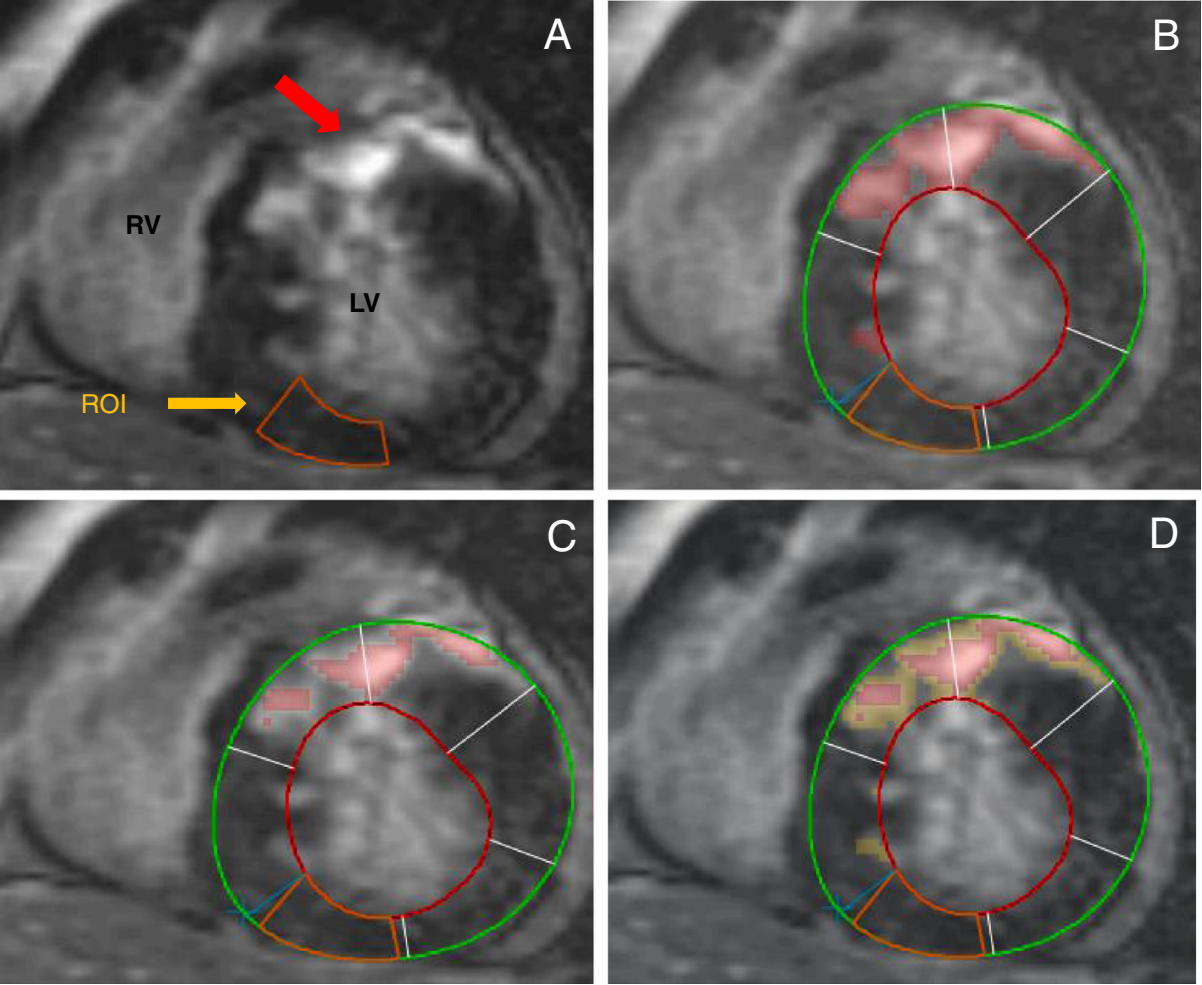
**Assessment of heterogeneous scar area (HSA). A)** Mid-ventricular short axis LGE image of a patient with considerable LGE (red arrow) in ventricular septum and anterior wall, and a region of interest in normal myocardium in the inferior wall (ROI; orange box) used to define thresholds for LGE. RV indicates right ventricle; LV, left ventricle. **B)** Endocardial (red) and epicardial (green) borders were outlined manually. Grayscale threshold 2SDs above the mean signal intensity of the ROI (red shading) was outlined. **C)** Grayscale threshold 4SDs above the mean signal intensity of the ROI (red shading) were considered as scar core and is shown in red shading. **D)** HSA_2-4SD_ defined as the signal intensity between ≥2SD and <4SD is shown in yellow shading, superimposed on scar core (>4SD) in red shading.

In order to identify the best SI threshold of scar detection as well as HSA, scar size was determined using a threshold of 4 or 6 SD above the mean of the remote normal myocardium and by manual tracing (Figure 1); HSA was defined as the difference between 2 SD and 4 SD (HSA_2-4SD_), between 2SD and 6SD (HSA_2-6SD)_ and between 4SD and 6SD (HSA_4-6SD_). The amount of LGE for each group was expressed in grams.

### ICD implantation and follow-up

All devices were implanted using standard surgical technique; choice of device was at the discretion of the implanting physician and the device was activated at completion of implantation.

All devices were programmed for both anti-tachycardial pacing (ATP) and shock with three zones of therapy including shock for ventricular fibrillation (VF), ATP followed by shock for fast VT, and a monitored zone for slower VT. Exact therapy settings were adjusted at the discretion of the implanting physician.

Devices were interrogated at 1 and 3 months after implantation and every 6 months thereafter in the device clinic. During regular follow-ups in the device clinic appropriate sensing was confirmed for all patients; the device was interrogated, and recorded events and ICD therapy reviewed. Stored electrograms prior to device therapy were assessed by an experienced cardiologist blinded to CMR findings. Appropriate ICD therapy was defined as antitachycardia pacing or shocks delivered for ventricular tachyarrhythmias [17-20].

### Statistics

Continuous variables are presented as mean ± SD. Categorical variables are presented in number and percentage. Pre-ICD implantation NYHA class is presented in median ± interquartile range of 25-75%. Survival time for appropriate ICD therapy outcome was defined to be the time (number of days) from ICD implantation to the appropriate ICD therapy. The end of the follow-up period was September 30, 2011. If the patient did not have an appropriate ICD therapy in the follow-up period, then the patient’s outcome was considered to be censored. Univariate Cox regression analysis was utilized to assess the association between each variable and the survival function of appropriate ICD therapy. A correlation matrix was made to assess the association among the predictors. Statistically significant variables from the univariate analysis were identified and entered into the Cox’s multivariable model as exploratory analysis only, due to the limited number of events. Variables such as HSA_2-4SD_ and HSA_4-6SD_ were both significant but were highly correlated; therefore, only one was entered into the multivariate model (HSA_2-4SD_ was more significant). Since HSA_2-4SD_ was found to be an independent predictor of appropriate ICD therapy, the study population was divided into 2 groups based on the median value of the HSA_2-4SD_ (5.9 g), and the event-rate of both cohorts was further analyzed by the method of Kaplan–Meier. The log-rank test was used to compare Kaplan-Meier survival curve. All statistical analyses were performed using SAS (v9.2, SAS Institute, Cary, NC). A P value of <0.05 was considered to be significant.

## Results

### Clinical and demographic data

A total of 41 patients who were referred for ICD implantation as the primary prevention of SCD and had a pre-ICD implantation LGE-CMR available, were identified. Baseline characteristics of the entire cohort are summarized in Table 1. One patient had a complicated ICD implantation which led to non-arrhythmic death 2 days after the procedure and was excluded from analysis. During the 25 ± 24 months follow-up, 4 patients died. Primary indication for cardiac MRI was assessment of cardiomyopathy (9 patients), LV and RV function (5 patients), and viability and scar assessment (26 patients).

**Table 1:** Patients demographics

	**Appropriate ICD therapy**	**No appropriate ICD therapy**	P-value*
	**n = 7**	**n = 33**	
Age (year)	66 ± 10	61 ± 11	0.824
Sex (M), n (%)	6 (86)	23 (70)	0.323
ICM, n (%)	5 (71)	15 (45)	0.427
BiV, n (%)	2 (28)	9 (27)	0.729
Diabetes, n (%)	5 (71)	9(27)	0.081#
Hypertension, n (%)	6 (86)	24 (72)	0.819
Dyslipidemia, n (%)	3 (43)	18(54)	0.233
Beta-blocker, n (%)	5 (71)	30 (91)	0.453
ACE-inhibitor, n (%)	7 (100)	31 (94)	0.996
Anti-arrhythmic, n (%)	0	2 (6)	0.995
Aspirin, n (%)	5 (71)	26 (79)	0.493
Pre-ICD NYHA	3 ± 1	3 ± 1	0.810
Inappropriate ICD therapy	0	2 (6)	0.996

BiV: Biventricular pacemaker; ICM: ischemic cardiomyopathy; ACE: angiotensin converting enzyme; Echo: echocardiography; NYHA: new york heart association.

* Univariate Cox’s proportional hazard model.

# was considered clinically significant and was entered into the multivar model.

### ICD follow-up data

After a mean follow-up of 25 ± 24 months (median = 13, IQR: 7–35 months), appropriate ICD therapy occurred in 7 (17.5%) patients including 2 patients who received ATP and 5 patients who received ICD shocks. One of the patients with ICD discharge underwent VT ablation. Two patients in the group without ICD therapy received inappropriate shocks for atrial fibrillation.

### CMR data

Data from LGE-CMR and function analysis are summarized in Table 2. There was a trend for greater scar size in the appropriate ICD therapy group by the 4 SD (P = 0.11) and 6 SD (P = 0.19) definitions. HSA was significantly greater in the appropriate ICD therapy group (HSA_2-4SD_, P = 0.001; HSA_4-6SD,_ P = 0.03) (Table 2).

**Table 2:** CMR measurements

	**Appropriate ICD therapy**	**No appropriate ICD therapy**	P-value*
	**n = 7**	**n = 33**	
LVM (g)	173 ± 30	160 ± 47	0.599
LVMI (g/m2)	91 ± 14	82 ± 20	0.369
LVEF (%)	23 ± 6.5	31 ± 9	0.04
LVEDV (ml)	263 ± 34	268 ± 107	0.831
LVEDVI (ml/m2)	140 ± 29	137 ± 45	0.559
LVESV (ml)	202 ± 35	195 ± 93	0.546
RVEDV (ml)	128 ± 19	160 ± 71	0.369
RVEDVI (ml/m2)	69 ± 12	78 ± 20	0.443
RVESV (ml)	66 ± 5.5	77 ± 41	0.719
RVEF (%)	48 ± 8	51 ± 12	0.332
LGE presence (%)	6 (86)	19 (57)	0.155
LGE4SD (g)	44 ± 29	27 ± 31	0.112
LGE6SD (g)	32 ± 25	21 ± 27	0.193
Visual (g)	34 ± 23	24 ± 28	0.245
HSA2-4SD (g)	17 ± 12	5 ± 7	0.001
HSA4-6SD (g)	11 ± 10	5 ± 7	0.038

LVM:Left ventricular mass; LVMI:left ventricular mass index; LVEDV:left ventricular end-diastolic volume; LVEDVI:left ventricular end-diastolic volume index; LVESV:left ventricular end-systolic volume; RVEDV:right ventricular end-diastolic volume; RVEDVI:right ventricular end-diastolic volume index; RVESV:left ventricular end-systolic volume; RVEF:right ventricular ejection fraction; LGE: late gadolinium enhancement; HSA:heterogenous scar area.

* Univariate Cox’s proportional hazard model.

# HSA2-4SD and HSA4-6SD were highly correlated (r=0.69, p-value: 0.0001), so only HSA2-4SD was entered into the multivariate model.

LVEF was also lower in the appropriate ICD therapy group (P = 0.04). The other functional and volumetric measurements were comparable between 2 groups (Table 2).

Univariate analysis showed a significant relationship between HSA and ICD therapy, whether it is defined by HSA_2-4SD_ (HR 1.11, chi-square = 10.73, p = 0.001), HSA_4-6SD_ (HR 1.09, chi-square = 4.28, p = 0.039), or HSA_2-6SD_ (HR 1.6, chi-square = 9.09, p-0.003). Due to the limited number of events, a multivariable Cox model was constructed as exploratory analysis to control for the possible confounding effects of LVEF and diabetes on the relationship between HSA_2-4SD_ and ICD therapy. HSA_2-4SD_ (HR_adjusted_1.08, 95%CI: 1.00-1.16, P = 0.04) remained a significant predictor of ICD therapy (Table 3), suggesting that for each gram increase in the HSA_2-4SD_, there is an 8% increase in the risk of appropriate ICD therapy.

**Table 3:** Multivariate Cox proportional hazard regression

	**HR**	**95% CI**	**P-value**
CMR-LVEF (%)	0.95	0.84-1.07	0.409
DM	2.61	0.39-17.49	0.321
HSA2-4SD (gr)	1.08	1.00-1.16	0.041

DM:diabetes mellitus; HSA:hetreogenous scar area; HR: hazard ratio.

**Figure 2:**
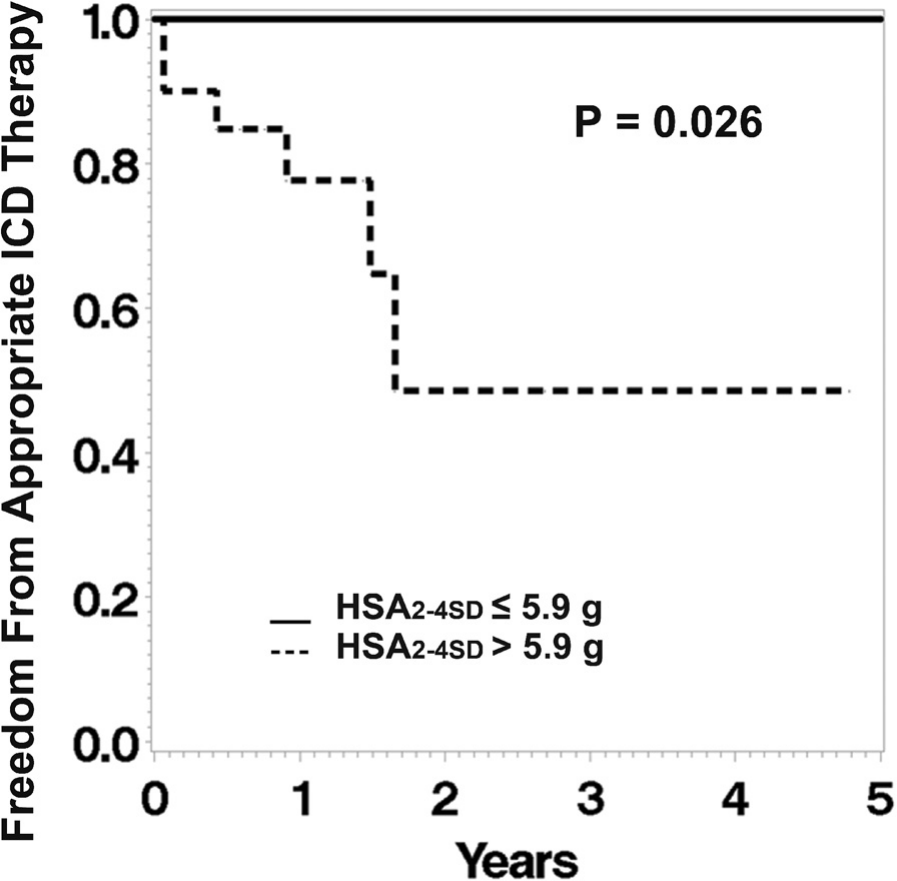
**Kaplan-Meier analysis shows that patients with HSA**_**2-4SD **_**greater than the median (5.9 grams) had lower survival free of appropriate ICD therapy (dashed line) whereas the patients with smaller HSA**_**2-4SD **_**(≤ 5.9 grams) had higher longer survival free of ICD firing (solid line).**

Kaplan-Meier analysis (Figure 2) showed that patients with HSA_2-4SD_ greater than the median (5.9 grams) had a lower survival free of appropriate ICD therapy (P = 0.026).

## Discussion

In this retrospective study of patients with ischemic and non-ischemic cardiomyopathy undergoing a CMR study prior to ICD implantation for primary prevention of SCD, we found that scar heterogeneity identified a subset of patients who subsequently had appropriate ICD therapy. Our data also showed a trend for LGE-CMR scar size as a predictor for ICD therapy. Additionally, in multivariate analysis, HSA was the only independent predictor which had significant impact on appropriate ICD therapy, while LVEF did not.

Current guidelines recommend ICD implantation for primary prevention of SCD based on LVEF thresholds [3,4,21]. Based on this metric, a large number of patients become eligible for this expensive treatment, but in a majority of patients receiving ICDs for primary prevention of SCD, appropriate ICD therapy does not occur during reported follow-up times [5]. Thus, there is a growing effort to better risk stratify patients with reduced LVEF most likely to benefit from prophylactic ICD implantation [6,7].

The major cause of SCD is ventricular tachyarrhythmia including VT and VF. Re-entry circuits appear to be a common mechanism underlying these ventricular arrhythmias [22] and the presence of myocardial scar has been indentified to be linked to the development of re-entrant arrhythmias [22,23]. LGE-CMR allows us to delineate myocardial fibrosis and scar tissue [24,25].

LGE presence has been shown to correlate with ventricular arrhythmia and ICD therapy in ischemic and non-ischemic cardiomyopathies [12]. In our study, the presence of scar was seen more frequently in patients requiring appropriate ICD therapy. However, 57% of patients who did not have ICD therapy had LGE.

In previous studies it has been shown that scar size in LGE-CMR [26] or in combination with electroanatomical mapping [27] can predict ventricular arrhythmia in patients with cardiomyopathy. Our data demonstrate not only that the extent of myocardial scar by different definitions (4SD, 6SD and visual thresholds) was larger in the appropriate ICD therapy group, but also the heterogeneity of scar is an independent predictor of appropriate ICD therapy. In previous studies, it has been reported that the extent of LV scar as well as peri-infarct zone were independent predictors of VT inducibility [15,28].

The partial volume effect is defined as an admixture of both infarcted (high SI) and noninfarcted (low SI) tissue. With averaging the 2 different SIs will be averaged, and those particular voxels will be represented by an intermediate SI (gray), affecting the HSA detection and measurement. The partial volume effect could result from volume-averaging effects of an area of uniformly fibrotic tissue (dense infarct scar) with an adjacent area of preserved, viable myocardium, particularly in situations in which spatial resolution is limited [24,28,29]. In this case, anatomically, there would be a narrow border between fibrotic scar and viable myocardium, and the limited spatial resolution would render an apparent intermediate SI in that border region. Partial volume effects due to averaging of normal and necrotic tissue have been demonstrated by LGE-CMR in experimental animal studies [24,29-31]. However, a second explanation is that the gray zone arises from the intermingling of preserved myocardium with bundles of fibrotic, infarcted scar within the same voxel. In this case, there would be a more gradual anatomic transition from dense, infarct core to preserved tissue beyond the infarct periphery. The latter mechanism is supported by pathological data [32,33].

We also wish to emphasize that having lower HSA does not invalidate previous risk stratification strategies based on other clinical factors. Decisions to exclude patients from receiving an ICD should not be based solely on HSA quantification, particularly in patients already deemed high risk prior to their CMR.

Our data in this study agree with previous studies. Schmidt and colleagues [15] studied 47 patients with a prior MI who underwent ICD implantation. They reported that infarct tissue heterogeneity was strongly associated with inducibility for monomorphic ventricular tachycardia, and was the single significant factor in a stepwise logistic regression.

To our knowledge, our study has the longest follow-up time and includes patients over a long time period from 2003 to 2011 which makes it unique among similar studies. Although some other studies have looked into a similar hypothesis, they all had a shorter follow-up time. Roes et al. showed that after a mean follow-up of 8.5 months, infarct gray zone was the strongest predictor of appropriate ICD therapy in patients with prior MI [34]. In addition, we have defined infarct heterogeneity by two different criteria to find the best metric that correlates well with appropriate ICD therapy. Scott et al. reported that during 19 months of follow-up of patients with coronary artery disease, appropriate ICD therapy occurred in patients with a greater scar size [35]. Iles et al. studied patients with cardiomyopathy and found that during 18 months of follow-up appropriate ICD therapy occurred more frequently in patients with LGE [12]. Compared to previous studies, which have mainly included ischemic populations, we have enrolled those with ischemic and non-ischemic cardiomyopathy.

A number of limitations of the current study need to be addressed. First, this study is a single center retrospective cohort and the results should be supported by a prospective multicenter trial. Second, the small sample size in this study prevented us from entering more variables into multivariable model, some of which may have potential confounding effects. Furthermore, the presented multivariable model is intended to be exploratory in nature, rather than demonstrating the independent relationship between HSA and ICD therapy. Also, most of the patients with appropriate ICD therapy had ischemic cardiomyopathy (71%). The sample size of the current study does not allow to study the value of LGE-CMR in CAD versus non-CAD patients. Thus, it is necessary to conduct a large trial in order to confirm the results of our study. In several patients with diffuse LGE in multiple regions of the myocardium, it was challenging to demarcate a region of interest (ROI) to define normal myocardium. However, great care was made to exclude regions of LGE and blood pool in the ROI, and all regions subsequently assigned as scar were verified visually before proceeding. In our study, 9 patients had 3D PSIR LGE images which were different from 2D LGE images. Despite this difference, we did not notice any difference in the result by excluding this subset of patients, presumably due to small size.

## Conclusions

In conclusion, LGE-CMR scar tissue heterogeneity is an important parameter for risk stratification of patients being considered for primary prevention ICD implantation. If supported by prospective, multicenter trials, LGE-CMR may emerge as the imaging metric for risk stratification for primary prevention ICD implantation.

## Competing interests

The authors declare that they have no competing interests.

## Authors’ contributions

HR: Conception and design, data extraction, analysis and interpretation of data, statistical analysis, drafting of the manuscript; AT: ICD data extraction; RHC: analysis and interpretation of data, statistical analysis, drafting of the manuscript SJP: ICD data extraction, THH: analysis and interpretation of data LN: analysis and interpretation of data, JLS: data extraction, SNH: data extraction, PJZ: Conception and design, AEB: Conception and design, interpretation of data, drafting of manuscript, MEJ: Conception and design, interpretation of data, drafting of manuscript, WJM: Conception and design, interpretation of data, drafting of the manuscript, RN: Conception and design, analysis and interpretation of data, drafting of the manuscript; All authors have given approval of this manuscript for publication.
